# Exploring the Landscape of Artificial Intelligence in Saudi Arabia's Healthcare Sector: Current Trends and Challenges

**DOI:** 10.7759/cureus.84163

**Published:** 2025-05-15

**Authors:** Mohammad Bayer, Amna Eisawi

**Affiliations:** 1 General Medicine, Glangwili General Hospital, Carmarthen, GBR; 2 Geriatrics, Tele-Geriatric Research Fellowship, Michigan State University, East Lansing, USA

**Keywords:** ai in healthcare, ai integration, artificial intelligence, healthcare innovation, saudi arabia

## Abstract

Artificial intelligence (AI) is advancing rapidly, and its potential to reshape the future of healthcare has been widely recognized. We conducted a review to understand the current state of AI in Saudi Arabia's healthcare sector. Our findings reveal that only 5.88% and 8.82% of major hospitals in Saudi Arabia have established specialized centers for AI and implemented AI in patient care, respectively. However, there has been a noticeable increase in academic interest, as evidenced by the growing number of research studies on the topic.

Saudi Arabia's Vision 2030 aims to position the country as a global leader in healthcare. Although institutions are gradually moving toward this goal, achieving it remains a distant prospect. Therefore, healthcare institutions and stakeholders must shift their approach from a consumer-oriented mindset to one driven by innovation and invention.

## Introduction and background

Artificial intelligence (AI) is a rapidly progressing and promising technology that is impacting many fields, including healthcare. While AI has been used and studied, although limitedly, since the 1950s, it became widely used in many industries only recently [1]. AI refers to the ability of machines to perform tasks that typically require human intelligence, such as learning, reasoning, and problem-solving. With advancements in machine learning (a subset of AI that allows systems to learn from data) and deep neural networks (computational models inspired by the human brain that are capable of recognizing patterns and making predictions), AI can perform complex tasks, including diagnostics and treatment planning. However, the healthcare sector presents unique challenges for AI due to stringent regulatory frameworks that prioritize patient safety, data confidentiality, and ethical considerations [2]. The nature of AI's continuous learning model, which necessitates large-scale data collection and sharing, poses significant risks to patient privacy, complicating its integration into this highly regulated field. For instance, the General Data Protection Regulation (GDPR) in Europe and the Health Insurance Portability and Accountability Act (HIPAA) in the United States have set strict guidelines for data handling, which AI systems must comply with to ensure patient trust and legal adherence [3]. These regulatory frameworks highlight the tension between innovation and privacy, a challenge that is particularly pronounced in healthcare settings where sensitive patient data is routinely processed [4].

Despite these challenges, AI has been successfully applied in various healthcare sectors where AI-driven applications like virtual wards, remote patient monitoring, and clinical decision support systems could potentially revolutionize patient care. For example, AI-powered diagnostic tools have demonstrated accuracy comparable to or exceeding that of human experts in fields such as radiology, pathology, and dermatology [5]. Moreover, AI has been instrumental in predicting patient outcomes, optimizing treatment plans, and reducing healthcare costs by streamlining administrative processes [6]. These advancements highlight the transformative potential of AI in healthcare, but they also underscore the need for robust frameworks to address ethical, legal, and social implications [7].

AI has been extensively explored in high-income countries, with the United States, Europe, and China at the forefront of leading AI research and development, making significant contributions to advancements in AI use in healthcare domains such as diagnostic imaging, predictive analytics, and robotic surgery [8]. For instance, the United States has invested heavily in AI research through initiatives like the National Artificial Intelligence Research and Development Strategic Plan, which aims to accelerate AI innovation in healthcare [9].

Most current literature focuses on AI applications in Western contexts, which often do not account for the unique healthcare infrastructure, regulatory environment, and cultural considerations present in Saudi Arabia. This review seeks to fill this gap by assessing the landscape of AI in Saudi Arabia's healthcare and exploring its progress and challenges.

## Review

Methods

In this narrative review article, we reviewed studies, institutional publications and reports, and official websites and social media accounts to select data focused on the application, regulation, and advancement of AI in Saudi Arabia's healthcare sector. Documentations were included regardless of publication date or language. Exclusion criteria consisted of non-Saudi-focused studies, studies with AI applications outside the healthcare sector, and publications that did not undergo peer review.

Firstly, we collected publicly available information about 34 hospitals from the "World's Best Hospitals - Saudi Arabia 2023" list by Newsweek [10]. Although the original list includes 35 hospitals, one hospital was excluded as it is exclusively focused on ophthalmology and does not provide general healthcare services, making it outside the scope of this review. Official websites and social media platforms of these hospitals were extensively reviewed to extract the number of hospitals with dedicated centers for AI research and application, the extent to which AI had been integrated into patient care, explicit referencing of AI in hospitals' mission statements, strategic plans, or summaries, and evidence of hospitals' participation in AI-related conferences.

Secondly, we reviewed publicly available information on the official websites and social media accounts of the Saudi Data and Artificial Intelligence Authority (SDAIA), the Saudi Commission for Health Specialties (SCFHS), the Ministry of Health (MOH), and the Saudi Food and Drug Authority (SFDA), in order to identify and summarize their currently disclosed initiatives and any publicly stated future directions regarding the integration of AI into healthcare operations.

Lastly, we analyzed the trends in AI publication frequency in Saudi Arabia over the period from 2019 to 2024 using advanced search terms with filters applied to restrict the title. We searched the Saudi Medical Journal and Journal of Healthcare Sciences for "Artificial Intelligence". Additionally, we searched Google Scholar, PubMed, and Cureus for "Artificial Intelligence" AND "Saudi Arabia" in the title. Studies were then sorted by year. It is important to note that we did not include the commonly used abbreviation "AI" as a standalone search term, which may have led to the omission of some relevant articles. This limitation could have impacted the comprehensiveness of our trend analysis and will be considered in future updates.

A researcher screened each record and assessed its eligibility based on the established criteria. Discrepancies in screening were resolved by consensus. No automation tools were used for this process, enhancing the accuracy of inclusion decisions. As this is a narrative review article, no formal risk of bias tool was employed. Instead, articles were assessed based on source credibility.

Results

After exploring official websites and social media accounts for the 34 major hospitals in Saudi Arabia, we found that only two (5.88%) have dedicated centers specifically focused on AI research and application. In direct patient care, AI has been implemented at least once in three hospitals (8.82%). Furthermore, only one hospital (2.94%) explicitly mentions "Artificial Intelligence" within their mission statement, vision, or strategic plans. Lastly, AI conference participation was noted for five hospitals (14.7%) (Table 1).

**Table 1:** Current state of AI research, implementation, and recognition in Saudi Arabia's leading hospitals as of 2023 AI: artificial intelligence

Activity	Number out of 34	Percentage
Presence of a specialized center for AI research and application	2	5.88%
Implementation of AI in patient care	3	8.82%
Clear mention of "Artificial Intelligence" in the hospital's summary, vision, or plans	1	2.94%
Participation in AI-related conferences	5	14.7%

The scope of AI initiatives and regulatory roles of four key Saudi Arabian government entities, the SDAIA, SCFHS, MOH, and SFDA, in the healthcare sector from their social media and official website are summarized in Table 2.

**Table 2:** Current AI circumstances from government authorities' perspective SDAIA: Saudi Data and Artificial Intelligence Authority; SCFHS: Saudi Commission for Health Specialties; MOH: Ministry of Health; SFDA: Saudi Food and Drug Authority; AI: artificial intelligence; NHCC: National Health Command Center

Authority	General roles and responsibilities	AI initiatives in healthcare
SDAIA	Leads national AI innovation	Established a Center of Excellence for AI applications in healthcare. Developed AI-based applications (Tawakkalna and Tabaud) for COVID-19, aiding in data monitoring, social distancing, and quarantine measures
SCFHS	Certifies healthcare professionals and regulates training	No AI initiatives or future plans identified on public platforms
MOH	Supervises all healthcare institutions. Regulates accreditations for healthcare institutions	Implements AI-based technologies such as Lunit INSIGHT CXR X-ray (Seoul, South Korea)analysis during the Hajj pilgrimage. Integrated AI into national initiatives like NHCC and "Seha" Virtual Hospital
SFDA	Regulates medications and medical devices	Issued a guidance for the use of AI in medical devices

The SDAIA is the national agency for AI innovation, supporting initiatives across multiple sectors. In healthcare, SDAIA's main contributions include establishing a Center of Excellence for AI applications and developing applications such as "Tawakkalna" and "Tabaud", which utilize AI for health data monitoring and were initially developed to aid in social distancing and quarantine during the COVID-19 pandemic [11].

The SCFHS plays a central role in certifying healthcare professionals and regulating their training standards [12]. No public records of explicit guidance or future plans for AI integration into the SCFHS were found.

On the other hand, the MOH has implemented the use of AI technologies such as the Lunit INSIGHT CXR system (Seoul, South Korea) for real-time X-ray analysis in Seha Virtual Hospitals. The initiative was used to aid in timely diagnosis, prioritizing critical cases, and enhancing patient care, particularly during high-demand periods like the Hajj pilgrimage [13]. In addition, the National Health Command Center (NHCC), an innovative collaboration between MOH and Ascend Solutions, leverages AI to monitor and analyze healthcare operations across Saudi Arabia. The NHCC was especially instrumental during the COVID-19 pandemic to provide MOH and other government entities with the required analytics, predictions, and recommendations to manage capacity and demand [14].

Finally, the SFDA, which is a regulatory entity of medications and medical devices, plays an integral role by issuing guidelines on AI use in medical devices, promoting safe and effective AI-driven healthcare solutions [15].

Upon reviewing research databases for AI in the healthcare sector in Saudi Arabia from 2019 to 2024, a notable progression was observed across PubMed, Cureus, and Google Scholar. In 2019 and 2020, there were no published research articles addressing AI's role in healthcare in the region. However, in 2021, two publications were listed in PubMed, while Google Scholar showed eight publications. In 2022, the number of publications remained low in PubMed (three publications), while Google Scholar saw a slight increase, with publications reaching 12.

**Figure 1:**
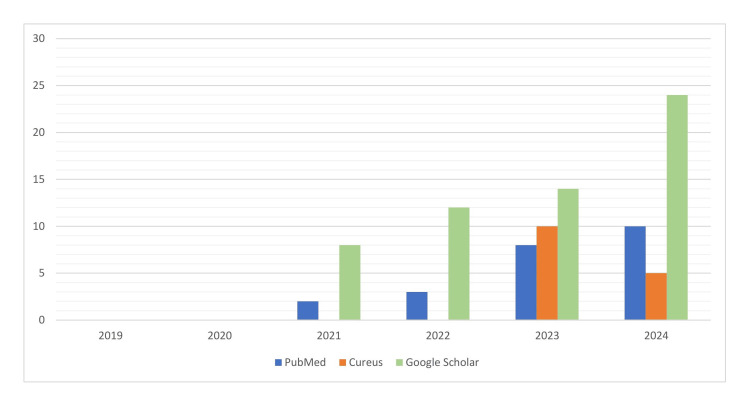
Number of research articles related to AI and healthcare in Saudi Arabia found in PubMed, Cureus, and Google Scholar databases from 2019 to 2024 AI: artificial intelligence

In 2023, the number of publications started to rise across all databases, with PubMed recording eight publications, Cureus showcasing 10, and Google Scholar 14. As illustrated in Figure 1, this upward trend persisted into 2024, with PubMed listing 10 publications, Cureus featuring five, and Google Scholar reaching 24.

**Figure 2:**
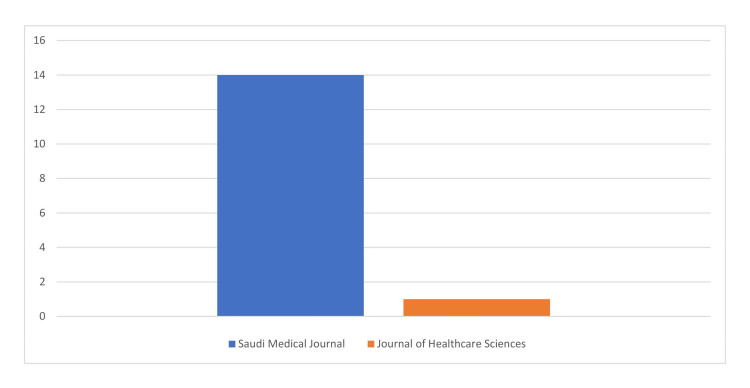
Number of research articles related to AI and healthcare in Saudi Arabia found in the Saudi Medical Journal and Journal of Healthcare Sciences AI: artificial intelligence

Upon further investigation of the academic interest in research related to AI in the healthcare sector in Saudi Arabia, as illustrated in Figure 2, a total of 14 research articles were identified in the Saudi Medical Journal, while two articles were found in the Journal of Healthcare Sciences.

Discussion

Although the relatively increasing number of hospitals' participation in AI conferences signals potential interest in AI advancements, the findings presented in Table 1 (which shed light on AI integration levels within the healthcare services of top Saudi hospitals) highlighted a limited active engagement and formal acknowledgment of AI's role in Saudi Arabia's top hospitals as of 2023. It also indicated that the practical integration of AI into clinical workflows is in its early stages, suggesting a relatively primitive AI infrastructure. Assuming the presence of sufficient finance and human resources, these could be the result of the lack of awareness of the promising potential of AI. This aligns with studies identifying insufficient institutional awareness and training as critical barriers to technological adoption in healthcare [16]. Despite robust evidence that AI-driven tools, particularly in radiology, demonstrate significant improvements in reducing diagnostic workloads (e.g., automating image analysis) and enhancing accuracy through pattern recognition, minimizing inter-observer variability, and expediting turnaround times [17,18], these benefits remain underutilized in many clinical settings. For instance, AI algorithms in mammography have shown a 20-30% reduction in false-positive rates while maintaining sensitivity, exemplifying its potential to refine diagnostic workflows [19].

Moreover, raising awareness must also address concerns among healthcare professionals about AI replacing human roles, a fear rooted in misconceptions about autonomy and job security. Empirical studies emphasize that AI functions optimally as a decision-support tool, enhancing physicians' capabilities by prioritizing complex cases, reducing cognitive fatigue, and mitigating diagnostic errors, roles that complement rather than displace clinicians [20,21]. Framing AI as a collaborative partner, rather than a competitor, fosters trust and preserves the irreplaceable physician-patient relationship, which remains central to ethical care delivery. Such reframing would accelerate the acceptance of AI integration into healthcare systems, not only at institutional levels but also among frontline providers, including clinicians and nurses, by aligning technological adoption with professional values and patient-centered outcomes [20,22].

In an effort to clarify how institutional and governmental activities together shape the current and future landscape of AI in Saudi Arabia's healthcare system, we provided an overview of AI-related initiatives and regulatory roles undertaken by major Saudi government authorities in the healthcare sector. The current scene highlights extensive AI involvement that goes beyond individual hospitals, demonstrating structured efforts at the governmental level to advance AI's role in healthcare. The MOH spearheads AI integration, with practical applications and projects highlighting its strong commitment [11,13]. Considering the burgeoning integration of AI within local governance, the role of government is pivotal. Governmental bodies are essential in steering the adoption of AI to ensure alignment with public values, equitable service provision, and the mitigation of potential risks [23]. Their involvement extends beyond mere facilitation, encompassing strategic direction to maximize societal benefit and cultivate responsible innovation in local government operations [23]. By actively participating, governments ensure that AI serves as a potent tool for enhancing public welfare, a crucial aspect explored through real-world examples [24].

Contrary to other entities, the SCFHS has demonstrated comparatively fewer initiatives in integrating AI into healthcare training. This presents a notable opportunity for growth, particularly considering the escalating influence of AI on medical education and healthcare delivery [6]. SCFHS, as the primary body responsible for postgraduate medical education and professional development in Saudi Arabia, holds a pivotal position in spearheading AI integration across healthcare professions [12]. Building upon the insights of Henning et al., who highlight AI's transformative capacity in areas such as clinical skills refinement and the creation of adaptive learning pathways, SCFHS could leverage these models to enrich its professional education frameworks [25]. For instance, AI-powered simulation tools could be incorporated into residency programs to offer realistic, risk-free environments for practicing complex procedures and diagnostic reasoning, thereby enhancing clinical competency [26]. By strategically bolstering AI literacy and practical skill-building initiatives, SCFHS can significantly improve the preparedness of the Saudi healthcare workforce to effectively navigate and contribute to increasingly AI-augmented healthcare settings [27]. This proactive approach is crucial not only for optimizing the adoption of AI technologies within Saudi Arabia's healthcare system but also for ensuring patient safety and maximizing the benefits of AI-driven advancements [25].

On another note, the scarce number of research related to AI in healthcare in Saudi Arabia until 2020 highlights a delayed onset of academic engagement in this field. Increasing national efforts to integrate AI within the healthcare infrastructure, possibly supported by strategic government initiatives and emerging institutional interest, could be the drive for the increased and accelerated academic interest since 2021, as evidenced by the growing number of studies. Similar to the Qatar case study, which exemplifies the pivotal role of research in advancing health systems through evidence-based initiatives in genomics, patient safety, and dementia to inform policymaking, necessitate targeted investment, and foster multi-sectoral collaboration, Saudi Arabia's recent surge in AI-focused research underscores the transformative potential of aligning academic inquiry with national healthcare priorities [28]. The observed concentration of AI articles in the Saudi Medical Journal suggests either a greater institutional receptiveness to AI within its purview or simply the journal's inherent thematic specialization or possibly a combination of both when contrasted with the Journal of Healthcare Sciences.

Our study revealed a lack of reported collaboration between major hospitals themselves or between major hospitals and government agencies with regard to advancing the use of AI in the healthcare sector. This lack of collaboration could be a result of the need for extensive resources and infrastructure to advance research in this rapidly expanding field. Inter-institutional collaboration has been identified as a key driver for AI innovation in healthcare systems globally, as demonstrated by the European Union's Horizon 2020 initiatives [29].

## Conclusions

To shape the future of healthcare, Saudi Arabian institutions must shift from being consumers of innovation to pioneers of AI-driven solutions. While academic interest in AI is expanding, its integration into clinical workflows remains limited, suggesting that tangible progress is still in its early stages. Prioritizing advanced AI research, fostering collaboration among hospitals, government entities, and private investors, and increasing investments in AI initiatives could not only address local healthcare challenges but also position Saudi Arabia as a global leader in AI-driven healthcare innovation. However, the study's reliance on publicly available data may have constrained the depth of insights, underscoring the need for broader stakeholder engagement in future research to fully capture the evolving AI landscape in healthcare.
